# Adipocyte-derived PDGF-D counteracts age-associated obesity through ameliorating lymphatic function

**DOI:** 10.3389/fendo.2026.1963378

**Published:** 2026-11-04

**Authors:** Lian Xu, Yuwen Cheng, Jiaqi Li, Yonghua Zhai, Jingrong Lin, Zebei Zhang

**Affiliations:** 1 Department of Cardiovascular Medicine, Shanghai Institute of Hypertension, National Center for Translational Medicine·Shanghai, State Key Laboratory of Medical Genomics, Shanghai Key Laboratory of Hypertension, Ruijin Hospital, Shanghai Jiao Tong University School of Medicine, Shanghai, China; 2 Department of Cardiology, Huadong Hospital Affiliated to Fudan University, Shanghai, China

**Keywords:** adipocyte, lymphatic vessel, middle-age, obesity, PDGF-D

## Abstract

Normal lymphatic function protects against obesity by preventing abnormal lymph leakage, which drives excessive adipocyte proliferation in adjacent tissues. Notably, platelet-derived growth factor D (PDGF-D) has been reported to play key roles in lymphangiogenesis. However, whether PDGF-D regulates age-associated obesity through modulation of lymphatic function remains unknown. In the study, we found that middle-aged mice exhibited greater susceptibility to high-fat diet (HFD)-induced obesity than young mice, accompanied by reduced lymphatic vessel density, impaired lymphatic function, and decreased PDGF-D expression in adipose tissue. By generating adipocyte-specific PDGF-D knockout and overexpression mice, we demonstrated that PDGF-D deficiency, despite having no direct effect on adipocytes, was sufficient to induce lymphatic dysfunction, which in turn exacerbated HFD-induced obesity in young mice. While adipocyte-specific PDGF-D overexpression restored lymphatic function and reversed HFD-induced severe obesity in middle-aged mice. Moreover, stimulating lymphangiogenesis via adeno-associated virus (AAV)-mediated delivery of an engineered VEGF-C variant (VEGF-C156S) successfully suppressed HFD-induced obesity in young adipocyte-PDGF-D knockout mice. *In vitro*, we found that adipocyte-derived PDGF-D promoted tube formation, proliferation, and the integrity of lymphatic endothelial cells (LECs), thereby preventing lipid permeation and subsequently suppressing adipogenesis. In conclusion, these findings demonstrate an uncovered mechanism by which adipocyte-derived PDGF-D protects against age-related obesity through regulation of lymphatic function.

## Graphical Abstract

**Figure:**
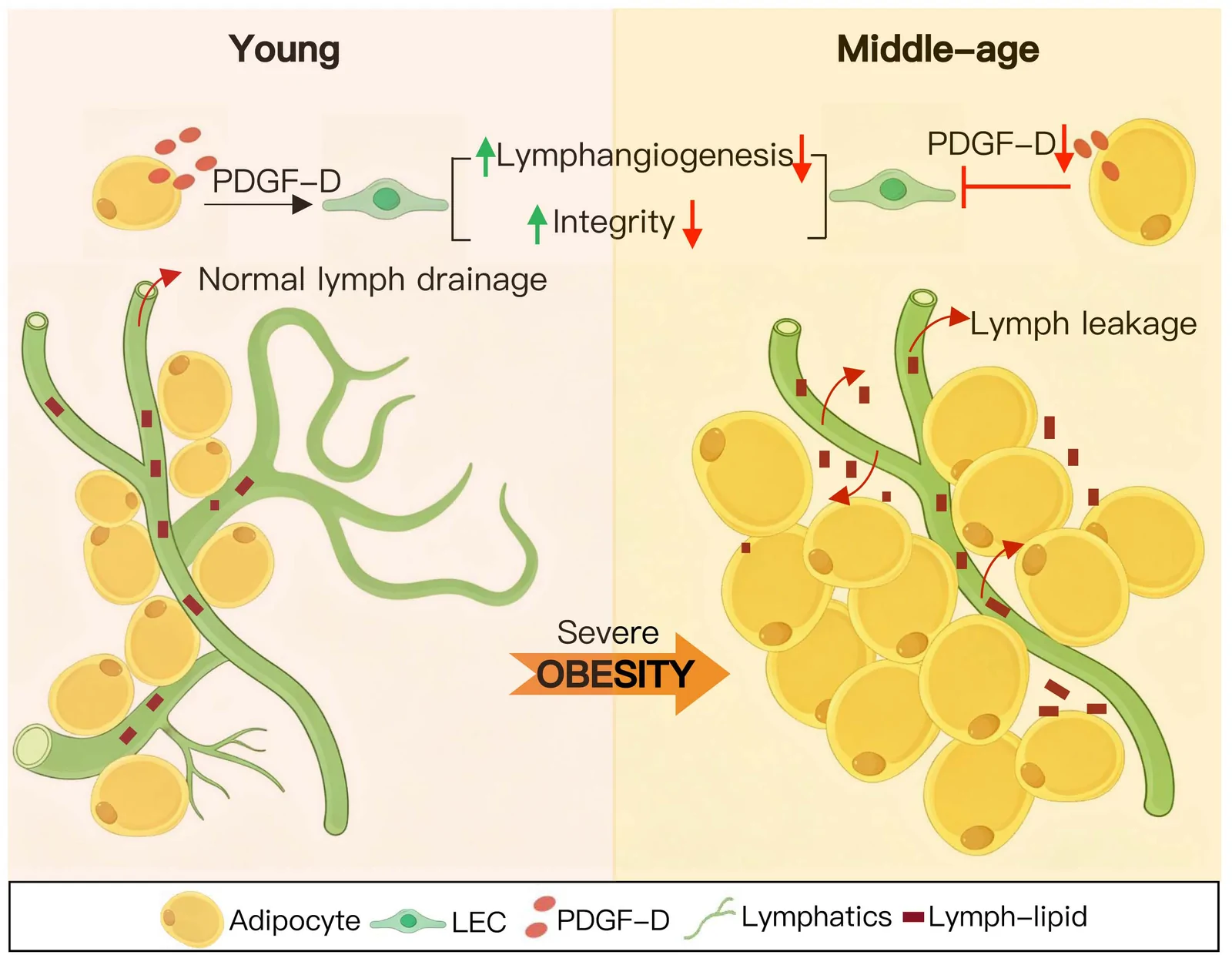

## Introduction

The prevalence of obesity is increasing worldwide and becoming one of the major health issues associated with morbidity and mortality (1–3). In the United States, data from the National Health and Nutrition Examination Survey (2021-2023) showed that the prevalence of obesity among adults was 40.3%, with the highest rate observed in adults aged 40–59 years (46.4%) (4). Similarly, a cross-sectional study of 422, 851 Chinese adults revealed that overweight and obesity rates are increasing most rapidly in the 40–49 years age group (5). These epidemiological data consistently demonstrate that middle-aged adults represent the population most affected by the obesity epidemic.

Lymphatic vessels are essential for lipid absorption and transport (6–11). Emerging evidence indicates that impaired lymphatic function in mouse models leads to lipid accumulation and subsequent obesity, as damaged lymphatic vessels cause lymph leakage, which drives adipogenesis in adjacent adipocytes (12, 13). Moreover, ageing has been shown to induce lymphatic dysfunction (14). For instance, age-related alterations in meningeal immunity have been demonstrated to impair lymphatic drainage function in the central nervous system (15). These findings suggest that age-related lymphatic dysfunction may play a causal role in obesity development.

Platelet-derived growth factor D (PDGF-D) is relatively new in the PDGF family and is known to be involved in lymphangiogenesis, cardiovascular remodeling, and organ fibrosis (16–19). Studies showed that PDGF-D activated fibroblasts, resulting in expansion of the lymphatic vasculature (20). However, whether PDGF-D participates in the regulation of lymphatic function and contributes to age-associated obesity has not been explored.

In the study, we found that middle-aged mice showed down-regulated PDGF-D expression in adipose tissue, accompanied by impaired lymphatic function. Therefore, we hypothesize that adipocyte-derived PDGF-D regulated age-associated obesity through modulation of lymphatic function. We created adipocyte-specific PDGF-D knockout (PDGF-DAT-KO) and overexpression (PDGF-DAT-Tg) mice and subjected them to high-fat diet (HFD)-induced obesity. Additionally, we administered adeno-associated virus (AAV) encoding VEGF-C variant (VEGF-C156S), a pro-lymphangiogenic factor, to PDGF-DAT-KO mice to rescue lymphatic function. By whole-mount immunostaining of lymphatic marker Lyve1 (Lymphatic Vessel Endothelial Hyaluronan Receptor 1) and light-sheet microscopy, we excluded macrophage signals and quantified lymphatic vessel density. Combining *in vivo* evaluation of lymphatic function and obesity phenotypes in genetically modified mouse models with *in vitro* co-culture assays of adipocytes and lymphatic endothelial cells (LECs), we systematically explored the regulatory role of adipocyte-derived PDGF-D in the progression of age-associated obesity.

## Methods

### Animals and animal care

PDGF-D flox mice was generated by inserting flox sites on both sides of the pdgf-d exon via gene targeting method (Cyagen Biotechnology Co., Ltd.). PDGF-D flox mice were then bred with Adipoq-Cre mice (obtained from The Jackson Laboratory) to produce Adipoq-Cre+; PDGF-D flox heterozygote mice. The heterozygote mice were bred with PDGF-Dflox/flox mice to produce PDGF-D^AT-KO^ homozygote and littermate Adipoq-Cre−; PDGF-Dflox/flox (control) mice. PDGF-D^AT-Tg^ mice were generated as previously described (31). Briefly, a mouse pdgf-d open reading frame was inserted into the PiggyBac transposon gene expression vector after the adipocyte protein-2 promoter. Then, transgenic mice were generated by vector microinjection to the zygote. PDGF-D^AT-Tg^ mice were crossed with WT C57BL/6J mice, and nontransgenic littermates were used as control. HFD-induced obese mice were fed a diet containing 60% kcal from fat (Research Diets, D12492) for 12 weeks. Mice were weighed weekly during HFD feeding, and the body weight for each mouse was recorded as the average of three measurements. All animals had the same C57BL/6J background and free access to water and corresponding diet. All animal experiments were conducted in accordance with guidelines approved by the Institutional Guidelines established by the Committee of Ethics on Animal Experiments at Shanghai Jiao Tong University School of Medicine.

### Statistical analysis

Sample size of each experiment was estimated by power calculations based on data from previous studies and preliminary experiments. Results are presented as mean ± SEM and analyzed using GraphPad Prism 8.4 (GraphPad Software). All data sets (n ≥ 6) were subjected to the Shapiro-Wilk test for the normality distribution analysis. Normally distributed data were analyzed using the unpaired, two-tailed Student’s t-test (for comparing two groups), or one-way analysis of variance (ANOVA) (one independent variable) with a *post hoc* Tukey’s test. P<0.05 was considered statistically significant.

## Results

### Middle-aged mice exhibit impaired lymphatic function and aggravated HFD-induced obesity

To investigate the age-dependent differences in lymphatic function and obesity, young (12-week-old) and middle-aged (12-month-old) mice were assessed for lymphatic vessels and fed an HFD for 12 weeks (**Figure 1A**). Whole-mount staining for the lymphatic marker Lyve1 (Lymphatic Vessel Endothelial Hyaluronan Receptor 1) and PDPN (Podoplanin) showed reduced lymphatic vessel density in subcutaneous adipose tissue (SAT), epididymal white adipose tissue (eWAT) and interscapular brown adipose tissue (iBAT) of middle-aged mice, indicating an age-related decline in lymphatic density (**Figures 1B, C**; **Supplementary Figures 1A, B**). Consistently, middle-aged mice exhibited defective lymphatic drainage, as evidenced by reduced Evans blue dye clearance in SAT and eWAT (**Figures 1D, E**). Data showed that middle-aged mice gained significantly more body weight than young mice (**Figure 1F**; **Supplementary Figure 1C**). EchoMRI quantification further confirmed that middle-aged mice accumulated greater total fat mass with HFD treatment (**Figure 1G**). Histological staining of SAT and eWAT revealed markedly enlarged adipocytes in HFD-fed middle-aged mice (**Figures 1H, I**). Collectively, these data demonstrate that middle-aged mice display lymphatic dysfunction and show increased susceptibility to HFD-induced obesity.

**Figure 1:**
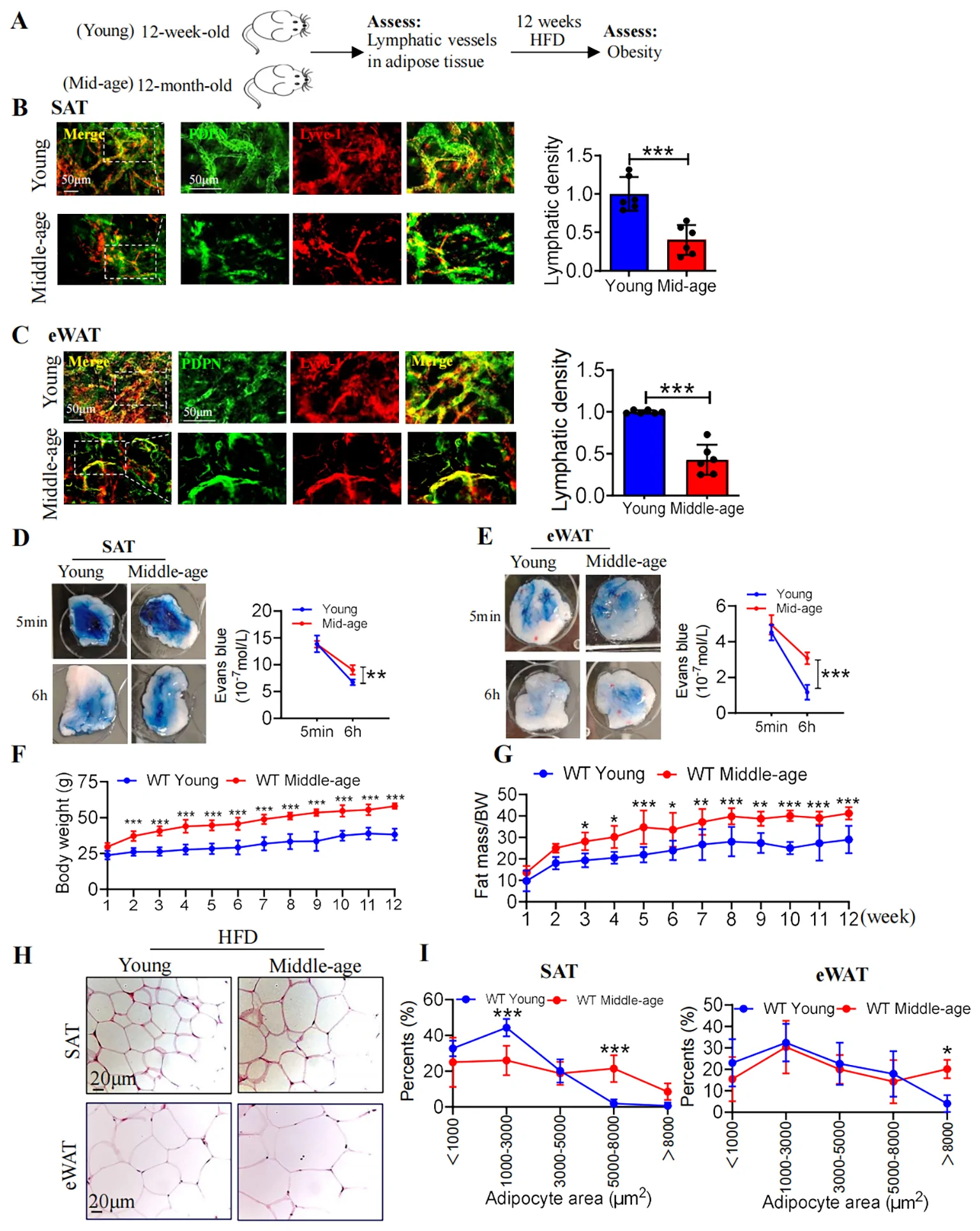
Middle-aged mice exhibit impaired lymphatic function and are susceptible to high-fat diet (HFD)-induced obesity. **(A)** Schematic diagram of the experimental design: chow-fed 12-week-old young and 12-month-old middle-aged mice were measured for lymphatic vessels and function, then fed a HFD for 12 weeks to induce obesity. **(B, C)** Whole-mount immunostaining for lymphatic vessel marker Lyvel and PDPN in subcutaneous adipose tissue (SAT) and epididymal white adipose tissue (eWAT) of young and middle-aged mice. The images in the first column are at lower magnification, while the images in the last three columns are enlarged views of the dashed boxes in the first-column images. **(D)** Lymphatic drainage function in SAT of young and middle-aged mice assessed by subcutaneously injection into the fat pad of 1% Evans blue (10ul) and quantification analysis of dye clearance. N=6/group. **(E)** Lymphatic drainage function in eWAT of young and middle-aged mice assessed by intraperitoneal injection of 1% Evans blue (10ul) and quantification analysis of dye clearance. N=6/group. **(F)** Body weight of young and middle-aged mice during the 12 weeks of HFD. N=10/group. **(G)** Fat mass measured by echoMRI. N=10/group. **(H)** H&E staining of SAT and eWAT of young and middle-aged mice after 12 weeks of HFD. **(I)** Quantification analysis of adipocyte size of SAT and eWAT. N=6/group. The n values are all biological replicates. *indicates P<0.05, **indicates P<0.01, ***indicates P≤0.001.

### PDGF-D expression is downregulated in adipose tissues of middle-aged mice

To determine whether PDGF-D contributes to age-related obesity and lymphatic dysfunction, we examined PDGF-D expression in multiple tissues of young and middle-aged mice. Quantitative real-time PCR (qPCR) showed that pdgf-d mRNA abundance was decreased in SAT and iBAT of middle-aged mice, while no significant age-related differences were detected in heart, aorta, and kidney (**Figure 2A**). Western blot quantification confirmed reduced PDGF-D protein levels in SAT and iBAT of middle-aged mice, with no alterations observed in non-adipose organs (aorta, kidney, and heart) (**Figures 2B-D**). Owing to abundant lipid droplets in eWAT that led to very low yield and poor quality of extracted RNA and protein, we used the more sensitive ELISA assay to measure PDGF-D levels in adipose tissues. Consistent with previous findings, the results showed that PDGF-D levels were decreased in SAT, iBAT, and eWAT from middle-aged mice (**Figure 2E**). Furthermore, we validated the changes in PDGF-D at the cellular level. Specifically, ELISA results revealed significantly decreased PDGF-D levels in adipocytes isolated from middle-aged mice, whereas no such changes were observed in isolated vascular smooth muscle cells (VSMCs) and bone marrow-derived macrophages (BMDMs) (**Figure 2F**). These results indicate that PDGF-D is suppressed in adipose tissue of middle-aged mice, implying a potential link between adipocyte-derived PDGF-D loss and age-associated obesity and lymphatic dysfunction.

**Figure 2:**
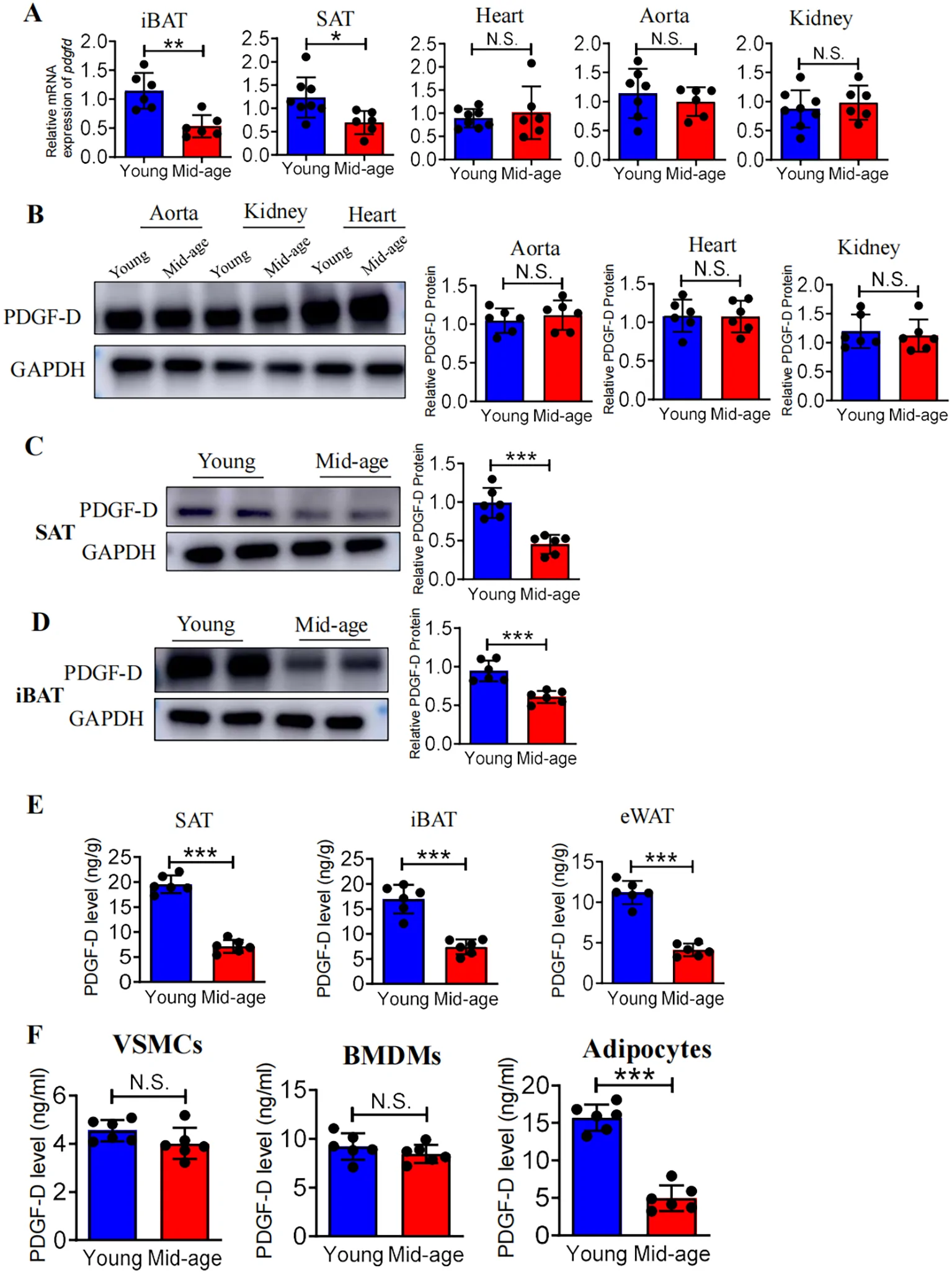
PDGF-D decreased in adipose tissue of middle-aged mice. **(A)** Quantitative real-time PCR analysis of *pdgf-d* genes in the iBAT, SAT, heart, aorta, and kidney tissues. N=6/group. **(B)** Western blot and quantitative analysis of PDGF-D and GAPDH in the aorta, kidney and heart tissues. N=6/group. **(C, D)** Western blot and quantitative analysis of PDGF-D and GAPDH in the SAT and iBAT. N=6/group. **(E)** PDGF-D levels were measured by ELIS assays in the SAT, iBAT and eWAT. N=6/group. **(F)** The PDGF-D levels in isolated vascular smooth muscle cells (VSMCs), bone marrow-derived macrophages (BMDMs) and adipocytes from young and middle-aged mice were measured by ELISA assays. N=6/group. The n values are all biological replicates. N.S. indicates nonsense, *indicates P<0.05, **indicates P<0.01, ***indicates P<0.001.

### Adipocyte-specific PDGF-D deletion exacerbates HFD-induced obesity and lymphatic defects in young mice

To verify the specific role of adipocyte-derived PDGF-D in obesity and lymphatic dysfunction, we generated adipocyte-specific PDGF-D knockout mice (PDGF-D^AT-KO^) and age-matched control littermates, all fed a 12-week HFD. The specific deletion of PDGF-D in PDGF-D^AT-KO^ mice was confirmed by qPCR and ELISA in multiple tissues (**Supplementary Figures 2A, B**). PDGF-D deficiency in adipocytes did not affect food intake or energy expenditure in mice (**Supplementary Figure 2C**). However, young PDGF-D^AT-KO^ mice displayed accelerated body weight gain and elevated total fat mass compared with control mice (**Figures 3A, B**; **Supplementary Figure 2D**). H&E staining of SAT and eWAT revealed robust adipocyte hypertrophy in young PDGF-D^AT-KO^ mice (**Figures 3C, D**). To determine whether PDGF-D directly acts on adipocytes to modulate obesity, we examined the proliferation and adipogenic differentiation of PDGF-D-deficient adipocytes. The results showed no effect of PDGF-D deficiency on these processes, indicating that adipocyte-derived PDGF-D affects obesity indirectly rather than via a direct adipocyte-targeted mechanism (**Supplementary Figures 2E, F**). Interestingly, whole-mount Lyve1 and PDPN staining illustrated a striking reduction in lymphatic vessel density in SAT, eWAT, and iBAT of young PDGF-D^AT-KO^ mice (**Figures 3E, F**; **Supplementary Figures 2G, H**). Evans blue clearance assays in subcutaneous fat pads and eWAT consistently demonstrated compromised lymphatic drainage efficiency in young PDGF-D^AT-KO^ mice (**Figures 3G, H**). These genetic loss-of-function data recapitulate the obesity and lymphatic defects observed in middle-aged wild-type mice, suggesting that PDGF-D deficiency in adipocytes is sufficient to induce lymphatic dysfunction and aggravate HFD-induced obesity in young mice.

**Figure 3:**
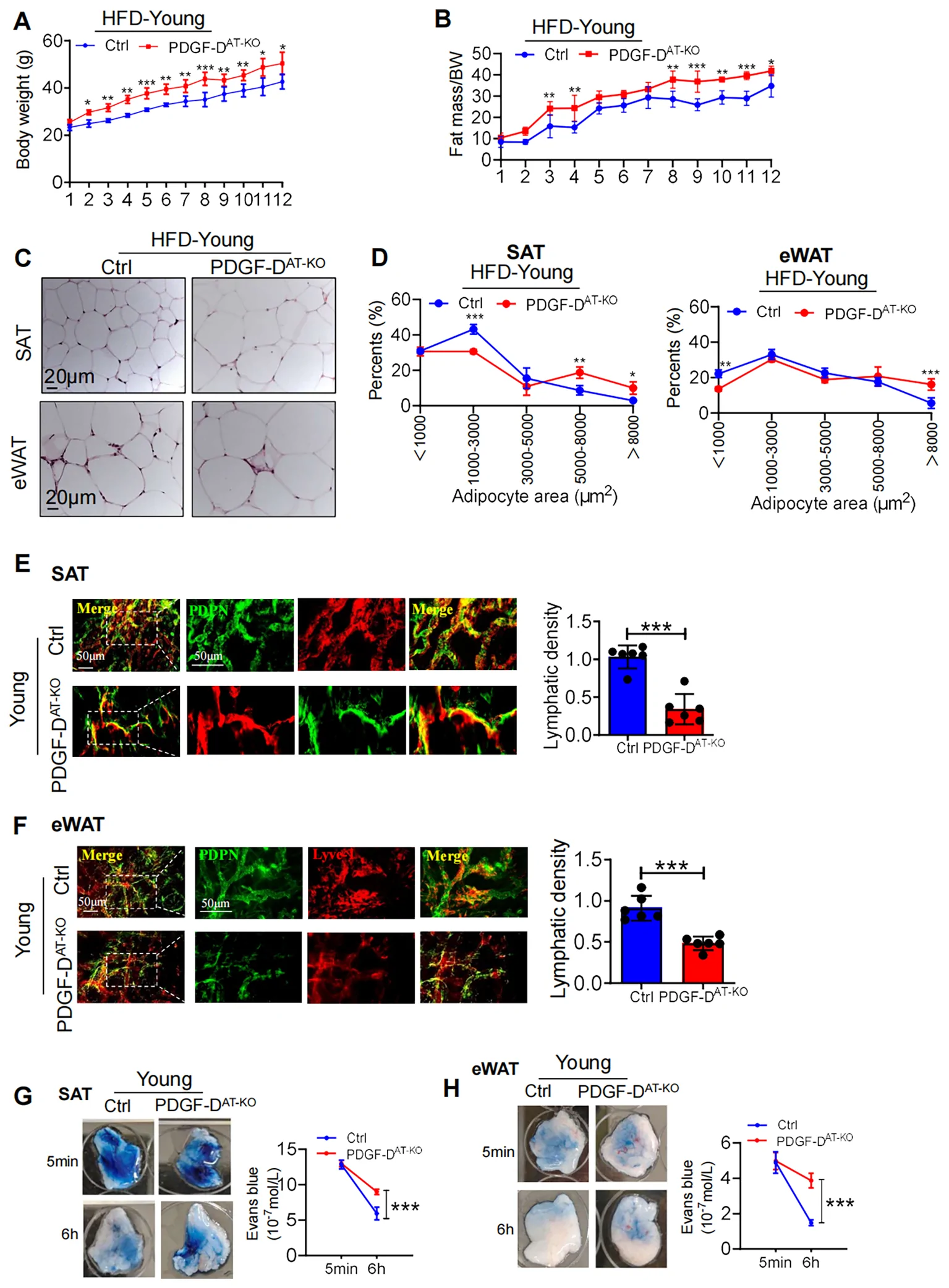
PDGF-D deficiency in adipose tissue accelerated obesity and induced lymphatic dysfunction in young mice. **(A)** Body weight of young control (Ctrl) and adipocyte-specific PDGF-D knockout (PDGF-D^AT-KO^) mice during the 12 weeks of HFD. N=10/group. **(B)** Fat mass measured by echoMRI. N=10/group. **(C)** H&E staining of SAT and eWAT of young control and PDGF-D^AT-KO^ mice after 12 weeks of HFD. **(D)** Quantification analysis of adipocyte size of SAT and eWAT. N=6/group. **(E, F)** Whole-mount immunostaining for lymphatic vessel marker Lyvel and PDPN in SAT and eWAT of young control and PDGF-D^AT-KO^ mice. N=6/group. **(G)** Lymphatic drainage function in SAT of control and PDGF-D^AT-KO^ mice assessed by subcutaneously injection into the fat pad of 1% Evans blue (10ul) and quantification analysis of dye clearance. N=6/group. **(H)** Lymphatic drainage function in eWAT of young control and PDGF-DAT-KO mice assessed by intraperitoneal injection of 1% Evans blue (10ul) and quantification analysis of dye clearance. N=6/group. The n values are all biological replicates. *indicates P<0.05, ** indicates P<0.01, ***indicates P≤0.001.

### Supplementation of VEGF-C156S by AAVs rescues lymphatic defects and mitigates severe obesity in young PDGF-D^AT-KO^ mice

We next explored whether lymphatic dysfunction plays the causal role in PDGF-D deficiency-mediated obesity. Young PDGF-D^AT-KO^ mice were randomly subjected to intravenous injection of either AAVs encoding a mutant VEGF-C (VEGF-C156S) that selectively drives lymphangiogenesis without inducing angiogenesis (21) (AAV-VEGF-C156S), or an empty control vector (AAV-NC). Eight weeks following AAV administration, all mice were fed a HFD for 12 weeks (**Figure 4A**). Whole-mount Lyve1 and PDPN staining of SAT, eWAT, and iBAT revealed that AAV-VEGF-C156S treatment significantly increased lymphatic vessels compared to AAV-NC controls (**Figures 4B, C**; **Supplementary Figures 3A, B**). Evans blue drainage assays confirmed restored lymphatic transport capacity in VEGF-C156S supplemented PDGF-D^AT-KO^ mice (**Figure 4D**). More importantly, AAV-VEGF-C156S-treated mice exhibited reduced body weight gain and lower total fat mass during HFD feeding (**Figures 4E, F**; **Supplementary Figure 3C**). Histological analysis of SAT and eWAT demonstrated smaller adipocyte size in the VEGF-C156S-treated group (**Figures 4G, H**). These data suggest that lymphatic restoration via VEGF-C156S supplementation counteracts PDGF-D deletion-mediated obesity.

**Figure 4:**
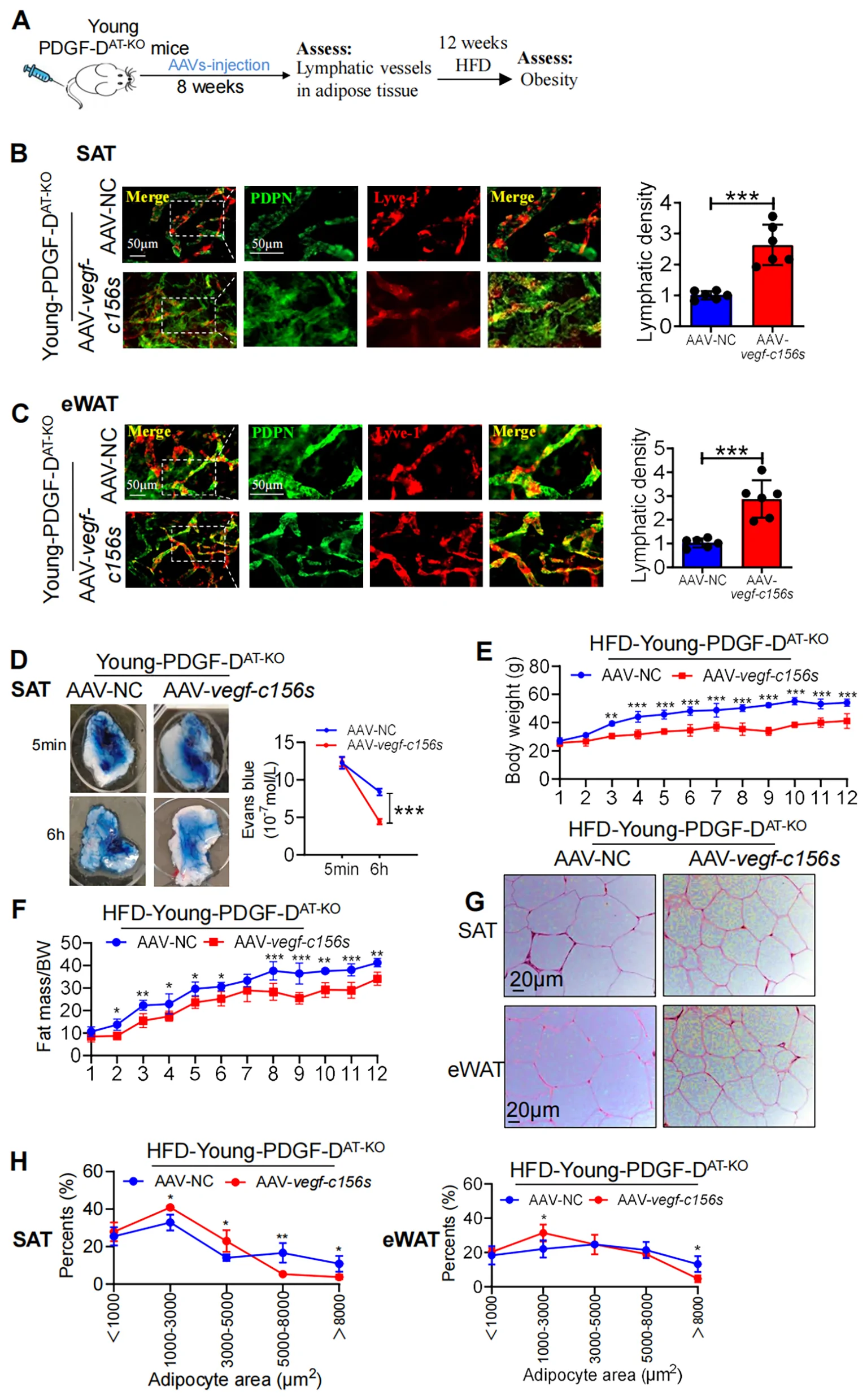
Restoration of lymphatic function via VEGF-C156S supplementation ameliorated obesity in HFD-fed young PDGF-D^AT-KO^ Mice. **(A)** Schematic diagram of the experimental design: chow-fed young PDGF-D^AT-KO^ mice were injected intravenously with AAVS encoding the *vegf-c156s* (AAV-*vegf-c156s*) or nonsense (AAV-NS) gene. Eight weeks post-injection, the mice were fed with a HFD for 12 weeks. **(B, C)** Whole-mount immunostaining for lymphatic vessel marker Lyvel and PDPN in SAT and eWAT of young PDGF-D^AT-KO^ mice with AAVS. N=6/group. **(D)** Lymphatic drainage function in SAT of young PDGF-D^AT-KO^ mice with AAVs assessed by subcutaneously injection into the fat pad of 1% Evans blue (10ul) and quantification analysis of dye clearance. N=6/group. **(E)** Body weight of young PDGF-D^AT-KO^ mice with AAVs during the 12 weeks of HFD. N=10/group. **(F)** Fat mass measured by echoMRI. N=10/group. **(G)** H&E staining of SAT and eWAT of young PDGF-D^AT-KO^ mice with AAVS after 12 weeks of HFD. **(H)** Quantification analysis of adipocyte size of SAT and eWAT. N=6/group. The n values are all biological replicates. *indicates P<0.05, **indicates P<0.01, ***indicates P≤0.001.

### Adipocyte-specific PDGF-D overexpression ameliorates lymphatic function and alleviates HFD-induced obesity in middle-aged mice

To confirm the protective effect of adipocyte-derived PDGF-D against obesity in the context of physiological aging, we utilized adipocyte-specific PDGF-D transgenic mice (PDGF-D^AT-Tg^) and middle-aged control littermates under 12-week HFD feeding. The overexpression of PDGF-D in PDGF-D^AT-Tg^ mice was confirmed by qPCR and ELISA in multiple tissues (**Supplementary Figures 4A, B**). Whole-mount Lyve1 and PDPN staining of SAT, eWAT and iBAT showed substantially higher lymphatic vessel density in PDGF-D^AT-Tg^ mice compared with age-matched controls (**Figures 5A, B**; **Supplementary Figures 4C, D**). Evans blue clearance assays in subcutaneous fat and eWAT demonstrated robust improvement in lymphatic drainage function of PDGF-D^AT-Tg^ mice (**Figures 5C, D**). Although PDGF-D overexpression in adipocytes did not affect food intake or energy expenditure of mice (**Supplementary Figure 4E**), PDGF-D^AT-Tg^ mice gained less body weight and accumulated less fat mass over the 12 weeks of HFD feeding (**Figures 5E, F**; **Supplementary Figure 4F**). SAT and eWAT histological staining revealed attenuated adipocyte hypertrophy in PDGF-D^AT-Tg^ mice (**Figures 5G, H**). Collectively, this gain-of-function genetic evidence confirms that elevating PDGF-D levels in adipocytes of middle-aged mice preserves lymphatic vasculature and drainage function, thereby limiting HFD-induced obesity.

**Figure 5:**
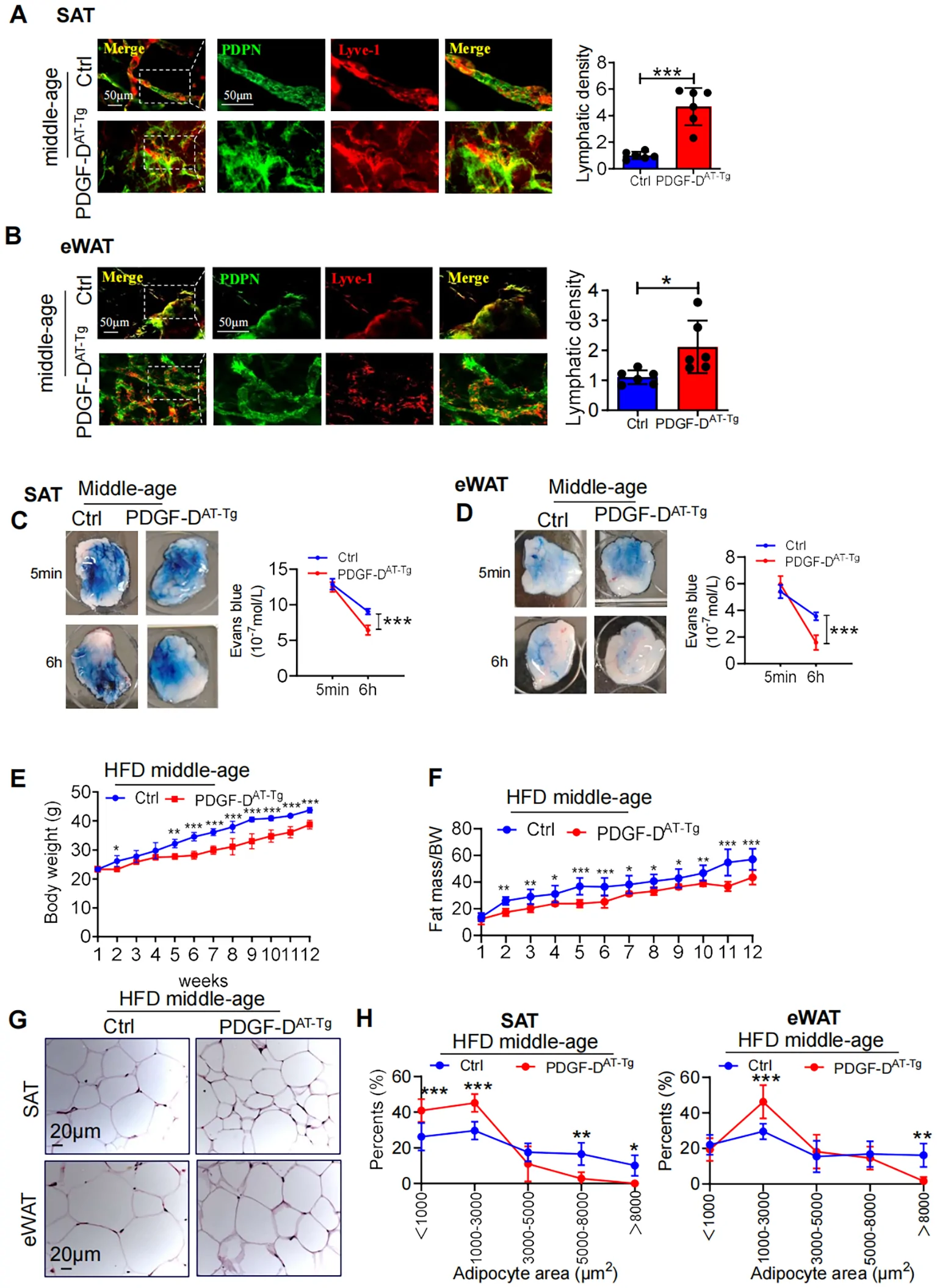
Adipocyte-specific PDGF-D overexpression rescued lymphatic function and counteracted HFD- induced obesity in middle-aged mice. **(A, B)** Whole-mount immunostaining for lymphatic vessel marker Lyvel and PDPN in SAT and eWAT of middle-aged control and adipocyte-specific PDGF-D overexpression mice (PDGF-D^AT-Tg^). N=6/group. **(C)** Lymphatic drainage function in SAT of middle-aged control and PDGF-D^AT-Tg^ mice assessed by subcutaneously injection into the fat pad of 1% Evans blue (10ul) and quantification analysis of dye clearance. N=6/group. **(D)** Lymphatic drainage function in eWAT of middle-aged control and PDGF-D^AT-Tg^ mice assessed by intraperitoneal injection of 1% evans blue (10ul) and quantification analysis of dye clearance. N=6/group. **(E)** Body weight of middle-aged control and PDGF-D^AT-Tg^ mice during the 12 weeks of HFD. N=10/group. **(F)** Fat mass measured by echoMRI. N=10/group. **(G)** H&E staining of SAT and eWAT of obese middle-aged control and PDGF-D^AT-Tg^ mice. **(H)** Quantification analysis of adipocyte size of SAT and eWAT. N=6/group. The n values are all biological replicates. *indicates P≤0.05, **indicates P≤0.01, ***indicates P≤0.001.

### Adipocyte-derived PDGF-D promotes lymphangiogenesis and lymphatic integrity *in vitro*

To confirm the direct regulatory effect of adipocyte-derived PDGF-D on lymphatic vessels, we co-cultured adipocytes and mouse lymphatic endothelial cells (mLECs) (**Figure 6A**). Compared to conditioned medium (CM) collected from wild-type young adipocytes, CM from middle-aged adipocytes, CM from young PDGF-D^AT-KO^ adipocytes, or CM from young adipocytes with PDGF-D neutralizing antibody failed to stimulate mLEC proliferation and tube formation. Whereas supplementation of recombinant PDGF-DD protein into middle-aged CM fully rescued mLEC proliferation and tube-forming capacity (**Figure 6B**). Transwell dextran permeability assays showed that CM from young adipocytes and exogenous PDGF-DD preserved the barrier integrity of mLECs, with only low levels of dextran detected in the lower chamber. In contrast, CM derived from middle-aged adipocytes, CM derived from PDGF-D-deficient adipocytes, or CM with PDGF-D neutralizing antibody failed to sustain mLEC barrier integrity, resulting in markedly elevated dextran permeation into the lower chamber (**Figure 6C**). Junctional morphology is also important for permeability of lymphatic vessels. Therefore, we have performed immunostaining of junctional protein VE-Cadherin in the transwell membrane. The results showed that the junctional morphology including button-like junctions, intermediate junctions, and zipper-like junctions did not change markedly among the different treatments, indicating that PDGF-D-mediated lymphatic function and permeability were mainly through the regulation of lymphangiogenesis but not junctional morphology (**Figures 6D, E**). Furthermore, we detected the permeability of oleic acid, which is a component of lymph and can promote adipogenesis (22), to assess mLEC integrity. The results showed that mLECs treated with CM from young adipocytes and exogenous PDGF-DD restricted oleic acid transport into the lower chamber, thereby suppressing lipid accumulation in pre-adipocytes. In contrast, CM from PDGF-D-deficient, CM from middle-aged adipocytes, or CM with PDGF-D neutralizing antibody exhibited impaired restriction of oleic acid transport, resulting in increased lipid accumulation in pre-adipocytes (**Figure 6F**). To exclude the aging effect of mLECs themselves, we compared the proliferation, tube-forming capacity, and barrier integrity in young and middle-aged mLECs. Accordingly, we termed mLECs in population doubling level (PDL)≤10 as “young cells” and mLECs in 20≤PDL<40 as “middle-aged cells” (23). Specifically, mLECs at passage 4 (P4) were considered young, and P16 mLECs were considered middle-aged. The findings showed no significant differences between young and middle-aged mLECs in lymphangiogenic capacity and barrier integrity (**Supplementary Figures 5A, B**). These data demonstrate that adipocyte-derived PDGF-D directly regulates mLECs, which promotes lymphangiogenesis, maintains barrier integrity, and thereby suppresses adipogenesis.

**Figure 6:**
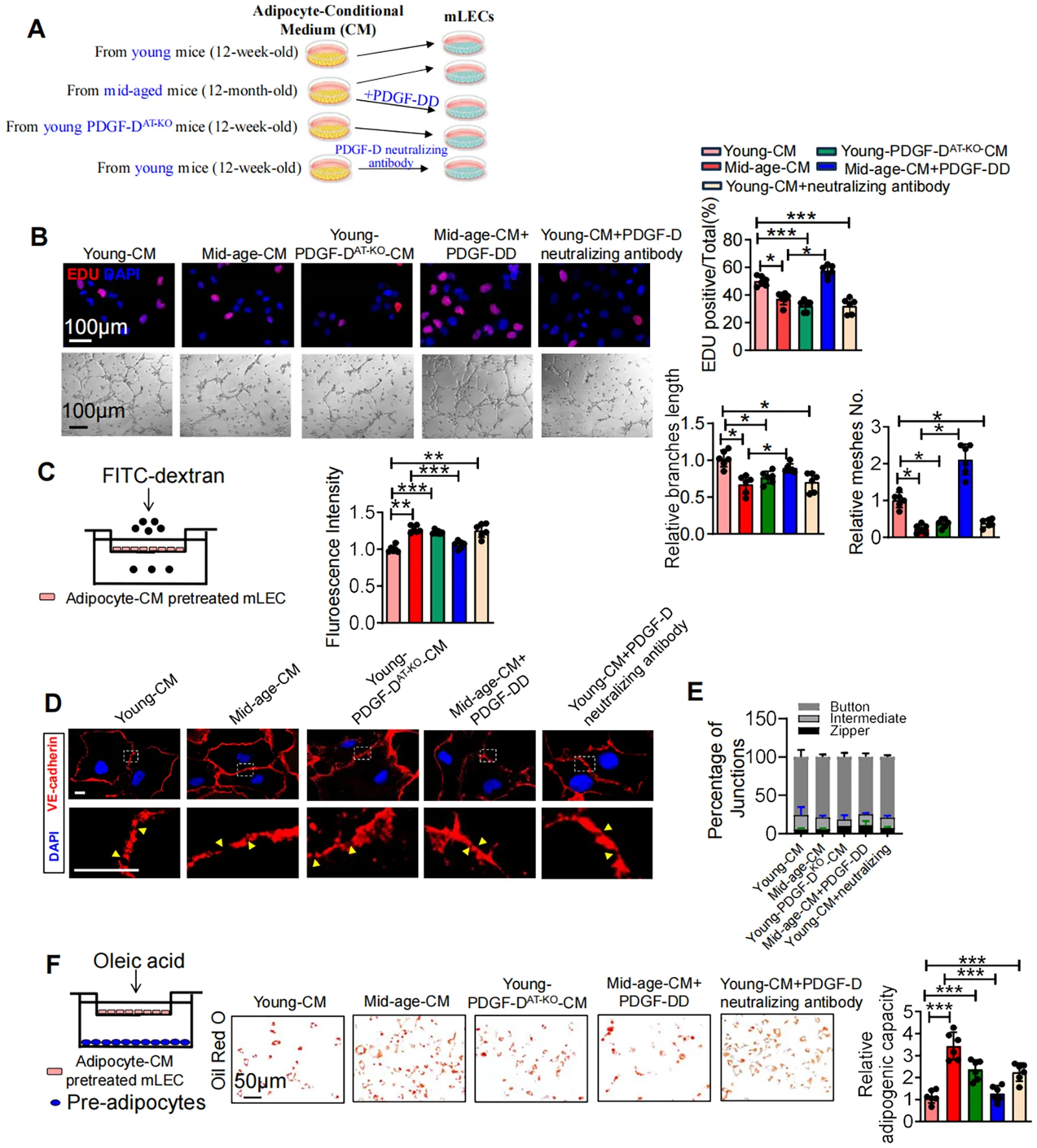
Adipocyte-derived PDGF-D promoted lymphangiogenesis and lymphatic integrity in vitro **(A)** Schematic diagram of the experimental design: conditional medium (CM) of cultured adipocytes from young, middle-aged, and young PDGF-D^AT-KO^ mice were collected and supplemented into mouse lymphatic endothelial cells (mLECs) cultures. Recombinant PDGF-DD protein (20uM) was added to PDGF-D^AT-KO^ CM to rescue the PDGF-D deficiency. Neutralizing antibody (10uM) was added to Young-CM to inhibit the effect of PDGF-D. **(B)** EdU (5-ethynyl-2'-deoxyuridine) proliferation assay, tube formation assay, and quantification analysis of mLECs stimulated by indicated adipocyte-CM. N=6/group. **(C)** mLECs treated with indicated adipocyte-CM were cultured in the upper chamber of a Transwell system. FITC-labeled dextran (1 mg/mL) was then added to the upper chamber, and quantified in the lower chamber 1 hour later. N=6/group. **(D)** The immunostaining of VE- Cadherin in the transwell membrane with indicated adipocyte-CM. **(E)** Quantification analysis of junctions were classified based on length: buttons (<5μm), intermediate junctions (5-11μm), and zippers (>11 μm). N=6/group. **(F)** mLECs treated with indicated adipocyte-CM were cultured in the upper chamber of a Transwell system and then transferred to wells containing pre-adipocytes in the lower chamber. After addition of oleic acid (100uM) to the upper chamber and 24 hours of co-culture, adipogenesis of the pre-adipocytes was measured by oil red o staining and quantified. N=6/group. The n values are all biological replicates. *indicates P≤0.05, **indicates P≤0.01, ***indicates P≤0.001.

## Discussion

The present study delineates a previously unrecognized mechanism whereby adipocyte-derived PDGF-D counteracts age-associated obesity by preserving lymphatic function. We systematically validated the causal cascade of aging-suppressed PDGF-D in adipose tissue, lymphatic dysfunction, and aggravated diet-induced obesity via genetic mouse models and cell co-culture assays. These findings provide mechanistic insights into the high prevalence of obesity in middle-aged populations and identify PDGF-D as a promising therapeutic target for age-related metabolic dysfunction.

In our study, middle-aged mice exhibited impaired lymphatic drainage and reduced lymphatic vessel density in the adipose tissues, which aligns with previous reports documenting age-related lymphatic dysfunction in the dura mater (24–26). However, our study extends these findings by identifying adipocyte-derived PDGF-D as a key molecular mediator of the age-related lymphatic decline. The downregulation of PDGF-D in adipose tissue of middle-aged mice, but not in non-adipose organs, suggests a tissue-specific vulnerability that may contribute to the increased obesity prevalence observed in middle-aged populations (27–29).

The important role of PDGF-D in obesity is firmly established through our adipocyte-specific knockout and overexpression mouse models (30, 31). Young PDGF-D^AT-KO^ mice recapitulated the obesity phenotype and lymphatic defects observed in middle-aged wild-type mice, confirming that loss of PDGF-D is sufficient to accelerate diet-induced obesity. Conversely, adipocyte-specific PDGF-D overexpression in middle-aged mice preserved lymphatic function and mitigated obesity development. Although adipocyte-derived PDGF-D has not affected food intake and energy expenditure, these reciprocal genetic manipulations provided compelling evidence that adipocyte-derived PDGF-D acted as a protective factor against age-associated obesity by maintaining lymphatic homeostasis. Another key finding of our study is that lymphatic dysfunction, but not the direct effect of PDGF-D on adipocytes, is the causal mechanism underlying PDGF-D deficiency-mediated obesity. The rescue experiment using AAV-mediated delivery of VEGF-C156S, a selective lymphangiogenic factor (32, 33), effectively restored lymphatic function in PDGF-D^AT-KO^ mice and attenuated severe obesity. Together, these results indicate a paracrine role for adipocyte-derived PDGF-D in obesity regulation.

Our *in vitro* studies further elucidate the basis of PDGF-D-related lymphatic homeostasis. Adipocyte-derived PDGF-D directly promoted LEC proliferation and tube formation, but did not alter the junctional morphology, indicating that PDGF-D acts as a paracrine signal from adipocytes to activate lymphangiogenesis. In addition, adipocyte-derived PDGF-D preserves LEC barrier integrity and restricts permeability to dextran and oleic acid, a function critical for obesity prevention, as impaired lymphatic integrity triggers lymph leakage and subsequent adipogenesis in neighboring adipose tissue (34–36).

A recent study showed that a newly emerged adipogenic progenitor population drives adipogenesis in middle-aged adipose tissue (37), while our study reveals that age-related PDGF-D decline causes lymphatic dysfunction and lipid leakage-driven adipogenesis. Together, these two complementary studies, addressing the mechanism at both the cellular and molecular levels, provide a comprehensive landscape of age-associated obesity.

It should also be noted that a limitation of the present study is that the available approaches for assessing lymphatic function remain relatively limited and heavily rely on dye-based drainage assays, such as Evans Blue. Although Evans Blue has been widely used to evaluate lymphatic drainage capacity (13, 21, 38), it primarily reflects the clearance of a single macromolecular tracer from the interstitial space rather than providing a comprehensive assessment of lymphatic function.

In conclusion, our study uncovers a previously unrecognized mechanism by which adipocyte-derived PDGF-D protects against age-associated obesity through ameliorating lymphatic function. The age-related decline in adipocyte-derived PDGF-D expression leads to lymphatic dysfunction, lipid leakage, and accelerated adipogenesis, thereby promoting obesity. These findings identify a crosstalk between adipocytes and LECs, provide adipocyte-derived PDGF-D as a potential therapeutic target for combating age-related obesity, and highlight the importance of maintaining lymphatic function for metabolic diseases.

## Data Availability

The original contributions presented in the study are included in the article/**Supplementary Material**, further inquiries can be directed to the corresponding author/s.
