# Supplementary material for: Adipocyte-derived PDGF-D counteracts age-associated obesity through ameliorating lymphatic function
Source: Front Endocrinol (Lausanne). 2026 Nov 4;17:1963378. doi: 10.3389/fendo.2026.1963378 (PMC13732135; doi:10.3389/fendo.2026.1963378)
Supplement: Supplementary file 1 [file SupplementaryFile5.pdf]

Method

Metabolic cage experiments

To examine the effect of the pdgf-d gene on the metabolism, metabolic phenotyping was carried out by using the PhenoMaster System (TSE Systems). Mice were placed individually into metabolic training cages to habituate to the new environment for 3 days. After that, the food intake (g) and the calorimetric parameters, including O2 consumption (VO2), CO2 production (VCO2) of the animals were continuously monitored for 24 hours. The energy expenditure (kcal/h) was calculated according to the Weir equation[1].

Fat Mass Measurement

Fat mass was determined using an EchoMRI-136 body composition analyzer for live small animals weekly during the HFD feeding.

Whole-mount Immunofluorescence Staining and Tissue Clearing

SAT, eWAT, and iBAT of mice were removed and fixed in 4% paraformaldehyde for 2 hours at room temperature and washed by phosphate buffer solution (PBS), then incubated with anti-Lyve-1 antibody (Abcam, ab14917) and anti-Podoplanin (PDPN) antibody (Abcam, ab319138) overnight at 4°C. Corresponding secondary antibodies labeled with AlexaFluor594 (Invitrogen) were applied. For tissue clearing, tissues were dehydrated with 20%、40%、60%、80% and 100% methanol for 1 hour respectively. After that the tissues were immersed with dichloromethane on a rotator at room temperature for 3 hours, followed by immersed with Benzyl Ether until images obtained. Specimens were viewed with light-sheet microscopy (Zeiss).

Lymphatic Functional Analysis

To evaluate the lymphatic drainage capacity in SAT and eWAT, 10 µL of 1% Evans blue dye diluted in 0.9% NaCl was injected either subcutaneously into the fat pad or intraperitoneally using a Hamilton syringe. Five minutes and six hours after dye injection, the adipose tissues were removed. The dye was eluted from equal amounts of dissected adipose samples with 2 mL formamide at 56 °C. The optical density was then measured by spectrophotometry (Biotrak II; GE Healthcare, Fairfield, CT) at 620 nm. The concentration in molarity was calculated as: molar concentration (mol/L) = [m_EB (g/g tissue) × tissue weight (g)] ÷ [molar mass (960.8 g/mol) × V_extract (L)].

Cell Culture

Mouse lymphatic endothelial cells (mLECs) were purchased from Cellbio (Shanghai) Bioengineering Co., Ltd. Cells were cultured in DMEM with 10% fetal bovine serum (FBS, Gibco, 16000-044) in 0.1% gelatin-coated culture dish or upper chamber of the Transwell system. When subcultures reached 80-90% confluence, serial passaging was performed by trypsinization, and the number of population doubling level (PDL) was monitored for further experiments. PDL was calculated as described previously[2]. Briefly, the number of population doublings (PD) that occurred between passages was calculated according to the equation PD=log2(CH/CS), where CH is the number of viable cells at harvest and CS is the number of cells seeded. We termed cells in PDLs ≤10 as “young cells” and cells in 20≤PDLs<40 as “middle-aged cells”. In the present study, mLECs at passage 4 (P4) were considered as young and P16 mLECs as middle-aged. Primary pre-adipocytes were isolated from subcutaneous adipose tissue as previously described[3]. Cells were differentiated into adipocytes in 10% FBS DMEM/F12 culture medium supplemented with an adipogenic cocktail (0.5 mM IBMX, 1 mM dexamethasone,1 mM rosiglitazone, 0.02 mM insulin, and 1 nM T3) for 48 hr, then were maintained in 0.02 mM insulin and 1 nM T3 and harvested at day 6-8 post-differentiation. BMDMs were isolated and cultured as previously described[3]. In brief, bone marrow was flushed from long bones and washed with PBS and then suspended in DMEM supplemented with 10% L-cell CM. L-cell CM were collected from L929 cells cultured in T 75-cm2 filter capflasks in DMEM for 10 days and filtered through 0.2-μm filters. 7 days later, all nonadherent cells were removed and the remaining cells split into 12-well and 24-well plates. Vascular smooth muscle cells (VSMCs) from mouse aorta are enzymatic digested accordingly[4]. Briefly, the aorta is excised, cleaned of connective tissue and adventitia, and cut into small pieces. The tissue fragments are then digested with a combination of enzymes (collagenase I and elastase). After centrifugation and filtration through a cell strainer to remove debris, the cells are resuspended in culture medium (DMEM supplemented with 10% FBS and antibiotics) and seeded onto culture dishes. The identity and purity of VSMCs are routinely confirmed by immunofluorescence staining for smooth muscle α-actin (α-SMA).

Hematoxylin-Eosin (H&E) Staining

Adipose tissues were fixed in 4% paraformaldehyde and embedded into paraffin, and then 5 um sections were prepared for H&E staining according to the manufacturer’s

instructions. The images were obtained by a laser-scanning confocal microscope (Zeiss). Images were analysed with ImageJ and Image-Pro Plus software.

Real-time qPCR

Total RNA was extracted from tissues and cells using TRIzol (Invitrogen) followed by chloroform extraction according to the manufacturer's protocol. RNA reverse transcription was performed using the PrimeScript RT Reagent Kit (Takara). Real-time quantitative polymerase chain reaction (qPCR) was performed with an ABI PRISM 7900HT system (Applied Biosystems). The primer used for PDGF-D was as follows: forward, ACGCGCTTGGAAAGTTTAGC; reverse, GTTGGTCTCTGAGGCTGCTT. Reactions were performed at 95°C for 30s followed by 40 cycles at 95°C for 5s and at 60°C for 30s. The relative expression of each gene was determined by normalizing to GAPDH.

Western Blot

Mouse tissues containing equal amounts of total protein were resolved by SDS-PAGE followed by immunoblot with primary antibodies PDGF-D (Invitrogen, 40-2100), and GAPDH (HRP-60004; Proteintech). The blots were probed with HRP-conjugated secondary antibodies, and the results of chemiluminescence were detected using an enhanced chemiluminescence detection system.

Cell Proliferation Assay

Cell proliferation assays were performed using Cell-Light EdU Apollo 594 In Vitro Imaging Kit (Ribobio Co., Ltd.). Briefly, we incubated live cells with 5-ethynyl-2′-deoxyuridine (EDU; 20 mM) for 1 h before fixation and stained nuclei according to the manufacturer's guidelines. Images were obtained by a laser scanning confocal microscope (Zeiss). Images were analyzed with ImageJ and Image-Pro Plus software.

Oil Red O Staining

To assess adipogenesis, cells were fixed with 4% formaldehyde for 15 minutes at room temperature. After fixation, cells were washed twice with PBS and then stained with 0.2% Oil Red O solution in 40% 2-propanol for 30 minutes at room temperature. Following staining, cells were washed thoroughly with distilled water to remove excess dye, and the stained lipid droplets were visualized under a light microscope (Zeiss). Images were analyzed with ImageJ and Image-Pro Plus software.

Tube Formation Assay

The mLECs were plated at a density of $5X10^4$ cells/well in 48-well plates previously coated with growth factor-reduced matrigel matrix (BD Bioscience,

356234). After 6 hours of incubation, tube formation was observed with a light microscope (Zeiss). Meshes number and branch length were counted in random microscopic fields from each well using ImageJ and Image-Pro Plus software.

Adeno-Associated Virus (AAV) Construction

For AAV viral vectors used in this study, the AAV9s expressing mouse VEGFC with a Cys156Ser mutation (AAV-VEGF-C156S), and control nonsense AAVs (AAV-NC) were custom-made by OBiO (Shanghai, China). Briefly, PCR-amplified fragments of mouse Vegfc coding sequence (NM_009506.2) with a Cys156Ser mutation were cloned into the pAAV-CMVeGFP-3FLAG backbone vector (CMV: cytomegalovirus promoter; eGFP: enhanced green fluorescent protein; 3FLAG: 3 copies of FLAG epitope tag) with self-cleaving 2A peptide (P2A) between sequences encoding VEGFC and eGFP (AAV-CMV-VEGFC-P2A-eGFP-3FLAG). AAVs were delivered intravenously through the tail vein at a dose of $1X10^{11}$ v.g. per mouse.

Permeability Assay

To evaluate lymphatic endothelial barrier integrity, mLECs were seeded onto the upper chamber of the Transwell inserts and cultured to a confluent monolayer. After starvation for 4 hours, FITC-dextran (70kDa, 1 mg/mL in PBS, Invitrogen D1823) or oleic acid (100uM, Merck 05508) were added to the upper chamber. For FITC-dextran permeability, aliquots were collected from the lower chamber 1 hour later, and fluorescence intensity was measured at Ex/Em 490/520 nm using the spectrophotometer. For oleic acid permeability, adipocytes in the lower chamber were subjected to Oil Red O staining 24 hours later to visualize lipid accumulation.

VE-cadherin immunostaining and Quantification of junction types

Cells were fixed in 4% paraformaldehyde for 15 min at room temperature, permeabilized by 0.1% Triton X-100 for 15 min and blocked in 1% BSA for 30 min, incubated with antibodies against VE-Cadherin overnight at 4℃ and fluorescent labelled-secondary antibodies for 2 hours at room temperature. The stained samples were mounted in fluorescent mounting medium and imaged using a Zeiss microscope. Buttons were considered short, discrete, unconnected sections of VE-cadherin protein. Zippers were considered long and continuous sections of VE-cadherin protein according to previous studies[5-7].

1.WEIR JB. New methods for calculating metabolic rate with special reference to protein metabolism. J Physiol. 1949 Aug;109(1-2):1-9.

2. Makpol S, Durani LW, Chua KH, Mohd Yusof YA, Ngah WZ. Tocotrienol-rich fraction prevents cell cycle arrest and elongates telomere length in senescent human diploid fibroblasts. J Biomed Biotechnol. 2011;2011:506171.
3. Cheng YW, Zhang ZB, Lan BD, Lin JR, Chen XH, Kong LR, Xu L, Ruan CC, Gao PJ. PDGF-D activation by macrophage-derived uPA promotes AngII-induced cardiac remodeling in obese mice. J Exp Med. 2021 Sep 6;218(9):e20210252.
4.Krawutschke C, Koesling D, Russwurm M. Cyclic GMP in Vascular Relaxation: Export Is of Similar Importance as Degradation. Arterioscler Thromb Vasc Biol. 2015;35(9):2011-2019.
5. Jannaway M, Iyer D, Mastrogiacomo DM, Li K, Sung DC, Yang Y, Kahn ML, Scallan JP. VEGFR3 is required for button junction formation in lymphatic vessels. Cell Rep. 2023 Jul 25;42(7):112777.
6. Zarkada G, Chen X, Zhou X, Lange M, Zeng L, Lv W, Zhang X, Li Y, Zhou W, Liu K, Chen D, Ricard N, Liao J, Kim YB, Benedito R, Claesson-Welsh L, Alitalo K, Simons M, Ju R, Li X, Eichmann A, Zhang F. Chylomicrons Regulate Lacteal Permeability and Intestinal Lipid Absorption. Circ Res. 2023 Aug 4;133(4):333-349.
7. Chou C, Paredes CC, Summers B, Palmer-Johnson J, Trivedi A, Bhagwani A, Hansen KB, Kristensen AS, Gyoneva S, Swanger SA, Traynelis SF, Reed HO. The thrombin receptor PAR1 orchestrates changes in lymphatic endothelial cell junction morphology to augment lymphatic drainage during lung injury. Nat Cardiovasc Res. 2025 Aug;4(8):964-975
